# Impact of acute rhythm restoration strategy on atrial fibrillation recurrence after cryoballoon ablation: a real-world cohort study

**DOI:** 10.1186/s12872-026-05753-5

**Published:** 2026-03-23

**Authors:** Yunus Emre Özbebek, Haluk Furkan Şahan, Mustafa Furkan Dursun, Yunus Öz, Engin Algül, Sinan İşcen, Hamza Sunman, Tolga Han Efe, Özcan Özdemir

**Affiliations:** Department of Cardiology, Ankara Etlik City Hospital, Ankara, 06170 Turkey

**Keywords:** Atrial fibrillation, Atrial fibrillation recurrence, Cryoballoon ablation, Direct current cardioversion, Left atrial diameter, Risk stratification

## Abstract

**Background:**

Acute rhythm restoration after cryoballoon ablation may reflect underlying atrial substrate; however, its independent prognostic value for atrial fibrillation (AF) recurrence remains uncertain.

**Objective:**

To evaluate the association between acute rhythm restoration strategy and echocardiographic parameters with AF recurrence after cryoballoon ablation in a real-world cohort.

**Methods:**

This retrospective single-center cohort included 395 consecutive patients undergoing first-time cryoballoon ablation. AF recurrence was defined as any atrial tachyarrhythmia occurring after a 3-month blanking period. Multivariable logistic regression and Cox proportional hazards analyses were performed to assess independent predictors.

**Results:**

During a median follow-up of 12 months, AF recurrence occurred in 23.9% of patients. In univariable analyses, successful sinus rhythm restoration after standard direct current cardioversion (DCCV) was associated with lower recurrence (*p* = 0.002); however, this association was not maintained after multivariable adjustment (OR 0.547, 95% CI 0.252–1.184, *p* = 0.126).

Left atrial diameter (LAD) demonstrated a borderline association with AF recurrence (OR 1.064 per mm increase, 95% CI 0.997–1.137, *p* = 0.063) and showed moderate discriminative ability (AUC 0.692; optimal cut-off ≈43.5 mm).

In multivariable Cox regression analysis, none of the examined variables were independently associated with time-to-recurrence.

**Conclusion:**

Acute cardioversion success was associated with recurrence in univariable analyses but was not independently predictive after multivariable adjustment. Left atrial diameter provided moderate but limited discrimination, supporting pragmatic risk stratification rather than a standalone predictor. These findings suggest that cardioversion success reflects the underlying atrial substrate rather than exerting a causal effect on long-term outcomes.

## Introduction

Cryoballoon ablation has become an established and widely adopted strategy for the treatment of atrial fibrillation (AF), offering favorable procedural efficiency and reproducibility compared with point-by-point radiofrequency ablation [1, 2]. Despite continuous technical refinements and growing operator experience, atrial fibrillation recurrence remains a substantial clinical challenge, particularly in real-world populations with a high burden of persistent AF and advanced atrial remodeling [3].

Identification of patients at increased risk for post-ablation AF recurrence is crucial for optimizing procedural strategies, tailoring follow-up intensity, and guiding adjunctive therapies. Previous studies have highlighted atrial structural remodeling, AF type, and disease duration as major determinants of arrhythmia recurrence after pulmonary vein isolation [4, 5]. However, many predictive models rely on complex imaging parameters or composite risk scores that may not be readily applicable in routine electrophysiology practice [6, 7]. Consequently, there is an ongoing need for simple, pragmatic markers that can be easily integrated into daily procedural decision-making.

Acute rhythm restoration during or immediately after ablation represents a potentially informative but underexplored procedural surrogate. Successful restoration of sinus rhythm, particularly following standard direct current cardioversion (DCCV), may reflect both the reversibility of atrial remodeling and the immediate electrophysiological response to ablation. Nevertheless, the prognostic significance of acute rhythm restoration strategies in the context of cryoballoon ablation has not been consistently evaluated, especially in heterogeneous real-world cohorts [4, 8–10].

Similarly, left atrial size remains one of the most accessible echocardiographic markers of atrial remodeling [11]. Although left atrial enlargement has been associated with AF recurrence, its discriminative performance and practical cut-off values in contemporary cryoballoon populations require further clarification, particularly when interpreted alongside procedural factors [4].

Therefore, the present study aimed to investigate the relationship between acute rhythm restoration strategies, simple echocardiographic parameters, and long-term AF recurrence in a large real-world cohort undergoing cryoballoon ablation. By focusing on readily available procedural and anatomical markers rather than complex predictive models, we sought to provide clinically applicable insights that may support risk stratification and post-ablation management in everyday electrophysiology practice.

## Methods

### Study design and population

This retrospective, single-center cohort study included consecutive patients who underwent first-time cryoballoon ablation for atrial fibrillation between January 2023 and December 2025 at Ankara Etlik City Hospital. The study protocol was approved by the institutional ethics committee (AEŞH-BADEK2-2025-772) and conducted in accordance with the Declaration of Helsinki.

Patients aged ≥ 18 years with paroxysmal or persistent atrial fibrillation undergoing index cryoballoon ablation were included. Patients with prior left atrial ablation or incomplete procedural or follow-up data were excluded.

### Ablation procedure

All procedures were performed using second-generation cryoballoon systems, including Arctic Front Advance (Medtronic) and POLARx (Boston Scientific) platforms, according to operator preference and institutional availability.

Procedures were performed under fluoroscopic guidance following transseptal access. Pulmonary vein isolation was confirmed using a circular mapping catheter, and no three-dimensional electroanatomical mapping system was routinely used.

Intraprocedural imaging was performed using fluoroscopy alone.

After completion of pulmonary vein isolation, direct current cardioversion (DCCV) was performed in patients who remained in atrial fibrillation. If sinus rhythm was not achieved, sequential electrical cardioversion attempts were performed.

### Definition of acute rhythm restoration

Successful acute rhythm restoration was defined as the presence of sinus rhythm at the end of the procedure, achieved by electrical cardioversion (including sequential attempts if required).

### Echocardiographic assessment

Transthoracic echocardiography was performed prior to ablation using commercially available ultrasound systems by experienced cardiologists according to current guideline recommendations. Measurements were obtained following the recommendations of the American Society of Echocardiography (ASE) [12].

Left atrial diameter (LAD) was measured in the parasternal long-axis view at end-systole using the leading-edge to leading-edge technique. Left ventricular end-diastolic diameter and left ventricular ejection fraction were also recorded using standard two-dimensional echocardiographic methods.

### Follow-up and outcome definition

Patients were followed through outpatient visits and clinical records. Follow-up duration was recorded in months.

A 3-month blanking period after ablation was applied.

The primary outcome was atrial fibrillation recurrence, defined as any documented atrial fibrillation or atrial tachyarrhythmia episode occurring after the blanking period, detected by:

- 12-lead ECG
- Holter monitoring
- or clinically documented symptomatic arrhythmia

### Post-procedural medical therapy

After the procedure, oral anticoagulation (vitamin K antagonist or direct oral anticoagulant) was prescribed according to CHA_2_DS_2_-VASc score.

Antiarrhythmic drug therapy, including amiodarone, propafenone, and beta-blockers, was prescribed at the discretion of the treating physician.

### Statistical analysis

Continuous variables were expressed as mean ± standard deviation or median (interquartile range), as appropriate. Categorical variables were expressed as counts and percentages.

Comparisons between groups were performed using Student’s t-test or Mann–Whitney U test for continuous variables and χ^2^ or Fisher’s exact test for categorical variables.

Univariable logistic regression analysis was used to identify potential predictors of AF recurrence. Variables with *p* < 0.10 in univariable analysis and clinically relevant variables were entered into multivariable logistic regression models.

To account for the time-dependent nature of AF recurrence, multivariable Cox proportional hazards regression analysis was also performed, and results were reported as hazard ratios (HR) with 95% confidence intervals. For time-to-event analysis, clinically relevant covariates were additionally considered in the Cox proportional hazards model.

Multicollinearity was assessed using variance inflation factors (VIF).

Receiver operating characteristic (ROC) analysis was used to evaluate the discriminative ability of left atrial diameter.

A two-sided *p* value < 0.05 was considered statistically significant. All analyses were performed using SPSS version 29.0 (IBM Corp., Armonk, NY, USA).

## Results

### Study population and baseline characteristics

A total of 395 patients who underwent cryoballoon ablation for atrial fibrillation were included in the study. During follow-up, AF recurrence was documented in 94 patients (23.9%), whereas 299 patients (76.1%) remained free from recurrence. Of the initial cohort of 395 patients, follow-up data were available for 393 patients, who constituted the study population for recurrence and outcome analyses. Baseline demographic, clinical, and echocardiographic characteristics of the study cohort are summarized in Table 1.

**Table 1:** Baseline demographic and clinical characteristics of the study population

Variable	Total population
Number of patients	395
Age, years	65 (58–70.5)
Male sex, n (%)	196/393 (49.9)
Body mass index, kg/m^2^	29 (28–31)
Atrial fibrillation type, n (%)	
Paroxysmal AF	153/391 (39.1)
Persistent AF	238/391 (60.9)
AF duration, n (%)†	
< 12 months	164/238 (68.9)
≥ 12 months	74/238 (31.1)
Left atrial diameter, mm	41.61 ± 5.65
Left ventricular end-diastolic diameter, mm	48 (45–51)
Left ventricular ejection fraction, %	60 (55–60)
Systolic pulmonary artery pressure, mmHg	30 (24–35)
Follow-up duration, months	12 (10–16)
Length of hospital stay, days	1 (1–2)
Medications, n (%)	
Beta-blocker	343/393 (87.3)
Calcium channel blocker	98/393 (24.9)
RAS blocker (ACEi/ARB)	217/393 (55.2)
Statin	227/393 (57.8)
Amiodarone	254/393 (64.6)
Propafenone	128/393 (32.6)
Mineralocorticoid receptor antagonist	53/393 (13.5)
Furosemide	57/393 (14.5)
Vitamin K antagonist	99/393 (25.2)
Direct oral anticoagulant	290/393 (73.8)
Antiplatelet therapy	46/393 (11.7)

Values are presented as mean ± standard deviation, median (interquartile range), or number (percentage), as appropriate. Percentages were calculated based on available (non-missing) data, and denominators reflect the number of patients with available data for each variable

*AF* atrial fibrillation, *RAS* renin–angiotensin system

^†^AF duration was available for 238 patients and was categorized as < 12 months or ≥ 12 months

### Procedural characteristics and clinical outcomes

Procedural characteristics and early clinical outcomes are presented in Table 2. Restoration of sinus rhythm after standard direct current cardioversion (DCCV) was achieved in the majority of patients, while a smaller subset required sequential DCCV attempts. Veno-occlusive myocardial ethanol infusion (VOM-EI) was performed in a limited proportion of cases, and anatomical pulmonary vein variations were identified infrequently.

**Table 2:** Procedural characteristics and early outcomes

Variable	Total population
Procedural characteristics	
Standard DCCV with sinus rhythm achieved, n (%)	186/233 (79.8)
Sequential DCCV with sinus rhythm achieved, n (%)	41/47 (87.2)
VOM ethanol infusion performed, n (%)	16/393 (4.1)
Anatomical variation present, n (%)	32/392 (8.2)
Early outcomes	
Length of hospital stay, days	1 (1–2)
Follow-up outcomes	
AF recurrence, n (%)	94/393 (23.9)
Rehospitalization, n (%)	61/393 (15.5)
All-cause mortality, n (%)	6/393 (1.5)

Values are presented as median (interquartile range) or number (percentage), as appropriate. Denominators reflect available (non-missing) data for each variable

*DCCV* direct current cardioversion, *VOM* vein of Marshall, *AF* atrial fibrillation

During follow-up, AF recurrence occurred in 23.9% of patients. Overall all-cause mortality was low (1.5%). Rehospitalizations were most commonly related to AF recurrence or recurrence-associated reinterventions. The median length of hospital stay was 1 day (interquartile range: 1–2 days).

### Comparison between patients with and without AF recurrence

Comparisons between patients with and without AF recurrence are detailed in Table 3. Patients who experienced AF recurrence had a significantly larger left atrial diameter compared with those without recurrence (median 44 mm vs. 40 mm, *p* < 0.001). Persistent AF was markedly more prevalent in the recurrence group (92.6% vs. 50.8%, *p* < 0.001), and an AF duration of ≥ 12 months was strongly associated with recurrence (*p* < 0.001).

**Table 3:** Comparison of patients with and without atrial fibrillation recurrence

Variable	No recurrence (*n* = 299)	Recurrence (*n* = 94)	*p*-value
Age, years	64 (13)	65 (12.25)	0.36
Body mass index, kg/m^2^	29 (2)	30 (4)	**< 0.001**
Male sex, n (%)	153 (51.2)	43 (45.7)	0.36
Persistent AF, n (%)	151 (50.8)	87 (92.6)	**< 0.001**
AF duration ≥ 12 months, n (%)	26 (17.2)	48 (55.2)	**< 0.001**
Left atrial diameter, mm	40 (7)	44 (7)	**< 0.001**
Left ventricular ejection fraction, %	60 (5)	60 (10)	**0.01**
LV end-diastolic diameter, mm	48 (6)	49 (7.25)	0.18
Systolic pulmonary artery pressure, mmHg	30 (13)	32.5 (12.5)	**< 0.001**
Standard DCCV success, n (%)	129 (86.0)	57 (68.7)	**0.002**
Sequential DCCV success, n (%)	17 (81.0)	24 (92.3)	0.25
VOM ethanol infusion, n (%)	10 (3.3)	6 (6.4)	0.19
Amiodarone use, n (%)	180 (60.2)	74 (78.7)	**0.001**
Anatomical variation present, n (%)	20 (6.7)	12 (12.8)	0.15

Values are presented as median (interquartile range) or number (percentage), as appropriate. Continuous variables were compared using the Mann–Whitney U test. Categorical variables were compared using the chi-square or Fisher’s exact test, as appropriate

*AF* atrial fibrillation, *DCCV* direct current cardioversion, *VOM* vein of Marshall

Successful restoration of sinus rhythm after standard DCCV was associated with a significantly lower recurrence rate (*p* = 0.002). Amiodarone use was more frequent among patients with AF recurrence (*p* = 0.001). In contrast, sex, beta-blocker use, statin therapy, VOM-EI, and pulmonary vein anatomical variations were not significantly associated with AF recurrence.

### Univariable logistic regression analysis

In univariable logistic regression analysis, persistent AF, AF duration ≥ 12 months, left atrial diameter, lower left ventricular ejection fraction, higher systolic pulmonary artery pressure, amiodarone use, and successful sinus rhythm restoration after standard DCCV were significantly associated with AF recurrence. Age and body mass index were not significantly associated with recurrence, although body mass index showed borderline significance. Variables with *p* < 0.10 in univariable analysis, together with clinically relevant covariates, were considered for inclusion in the multivariable model. The results of the univariable regression analysis are presented in Table 4.

**Table 4:** Univariable logistic regression analysis for AF recurrence

Variable	OR	95% CI	*p*-value
Age (per year)	1.008	0.984–1.032	0.503
Body mass index, kg/m^2^	1.053	0.989–1.120	0.107
Persistent AF	12.017	5.384–26.823	< 0.001
AF duration ≥ 12 months	5.917	3.256–10.755	< 0.001
Left atrial diameter, mm	1.131	1.080–1.183	< 0.001
Left ventricular ejection fraction, %	0.973	0.951–0.996	0.023
Systolic pulmonary artery pressure, mmHg	1.036	1.013–1.059	0.002
Amiodarone use	2.446	1.418–4.221	0.001
Successful sinus rhythm restoration after standard DCCV	0.357	0.186–0.686	0.002

Values are expressed as odds ratios (OR) with 95% confidence intervals derived from univariable logistic regression analysis. Variables with *p* < 0.10 in univariable analysis, together with clinically relevant covariates, were considered for inclusion in the multivariable model

*DCCV* direct current cardioversion, *AF* atrial fibrillation

### Multivariable logistic regression analysis

Multivariable logistic regression analysis was performed to identify independent predictors of AF recurrence (Table 5). Variables entered into the model included left atrial diameter, systolic pulmonary artery pressure, amiodarone use, and successful restoration of sinus rhythm after standard DCCV. AF type was not retained in the final model due to quasi-complete separation and resulting model instability.

**Table 5:** Multivariable logistic regression analysis for atrial fibrillation recurrence

Variable	Odds Ratio (OR)	95% CI (Lower)	95% CI (Upper)	*p*-value
Left atrial diameter (mm)	1.064	0.997	1.137	0.063
Systolic pulmonary artery pressure (mmHg)	1.005	0.977	1.033	0.739
Amiodarone use	1.370	0.678	2.767	0.380
Sinus rhythm after standard DCCV	0.547	0.252	1.184	0.126

Model statistics: Omnibus χ^2^ = 15.91 (*p* = 0.003); Nagelkerke *R*^2^ = 0.091; Hosmer–Lemeshow goodness-of-fit *p* = 0.840

**Fig. 1:**
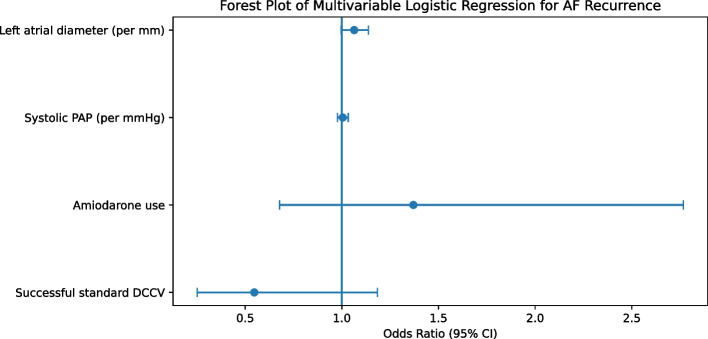
Forest plot of multivariable logistic regression analysis for atrial fibrillation (AF) recurrence. Adjusted odds ratios (OR) with 95% confidence intervals are shown for left atrial diameter, systolic pulmonary artery pressure, amiodarone use, and successful sinus rhythm restoration after standard direct current cardioversion (DCCV)

Left atrial diameter demonstrated a borderline independent association with AF recurrence (OR 1.064 per mm increase, 95% CI 0.997–1.137; *p* = 0.063). Systolic pulmonary artery pressure, amiodarone use, and successful standard DCCV were not independently associated with recurrence after adjustment. The model showed good calibration (Hosmer–Lemeshow *p* = 0.840). Adjusted odds ratios and confidence intervals are illustrated in Fig. 1

In multivariable Cox proportional hazards regression including age, sex, AF duration, left atrial diameter (LAD), standard DCCV success, hypertension, and diabetes, none of the covariates were independently associated with recurrence (overall model *p* = 0.525) (Table 6). AF type could not be retained in the model due to quasi-complete separation and collinearity with other variables.

**Table 6:** Multivariable Cox proportional hazards regression analysis for atrial fibrillation recurrence

Variable	Hazard Ratio (HR)	95% CI	*p*-value
Age (per year)	0.99	0.98–1.01	0.337
Male sex	0.80	0.61–1.05	0.112
AF duration ≥ 12 months	0.80	0.51–1.24	0.310
Left atrial diameter (per mm)	0.99	0.96–1.03	0.627
Successful standard DCCV	0.73	0.45–1.17	0.177
Hypertension	0.97	0.71–1.33	0.846
Diabetes mellitus	1.02	0.73–1.42	0.910

Values are expressed as hazard ratios (HR) with 95% confidence intervals derived from multivariable Cox proportional hazards regression. AF type was not included in the model due to quasi-complete separation and collinearity with other covariates. Overall model *p* = 0.525

### ROC curve analysis

**Fig. 2:**
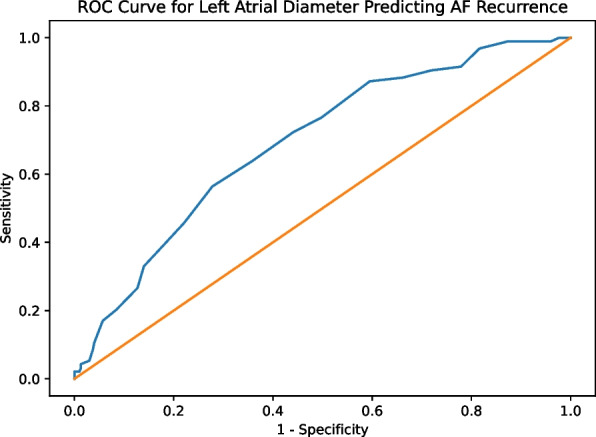
Receiver operating characteristic (ROC) curve of left atrial diameter for predicting AF recurrence. The area under the curve (AUC) was 0.692, indicating moderate discriminative ability. The optimal cutoff value was approximately 43.5 mm

Receiver operating characteristic curve analysis showed that left atrial diameter had moderate discriminative ability for predicting AF recurrence (AUC = 0.692, 95% CI 0.633–0.750; *p* < 0.001) (Fig. 2). A cut-off value of ≥ 43.5 mm provided the optimal balance between sensitivity (56.4%) and specificity (72.2%), corresponding to the highest Youden index.

### Complications and rehospitalization

**Fig. 3:**
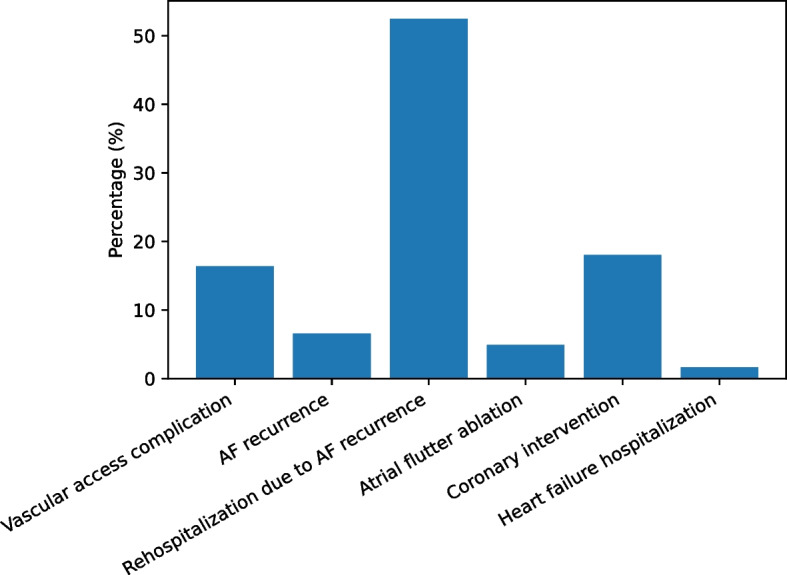
Distribution of procedure-related complications and causes of rehospitalization after cryoballoon ablation. Bars represent the percentage of patients experiencing each outcome category

Procedure-related complications and rehospitalization events are summarized in Fig. 3. Most patients did not require rehospitalization or experience major procedure-related complications during follow-up. Among patients requiring rehospitalization, AF recurrence was the most common cause, followed by recurrence-related reinterventions. Vascular access–site complications were infrequent, and other causes of rehospitalization—including atrial flutter ablation, coronary intervention, and heart failure–related hospitalization—occurred in a small proportion of patients.

### AF-free survival analysis

**Fig. 4:**
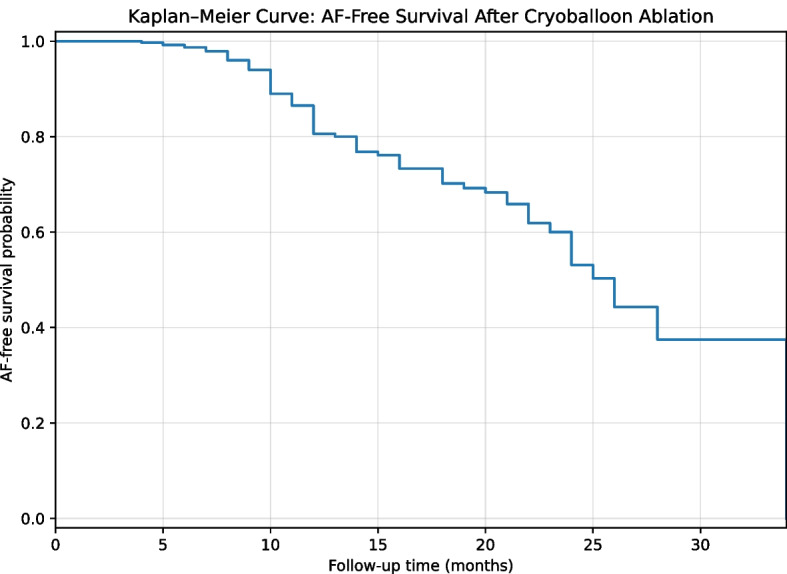
Kaplan–Meier curve of atrial fibrillation–free survival after cryoballoon ablation according to successful versus unsuccessful sinus rhythm restoration after standard DCCV

Kaplan–Meier analysis demonstrated a progressive decline in AF-free survival over the follow-up period (Fig. 4). The estimated mean AF-free survival time was 24.3 months (95% CI 22.8–25.9), while the median AF-free survival time was 26.0 months (95% CI 23.6–28.4), reflecting the presence of censored observations during follow-up. The estimated AF-free survival rate was 80.6% at 12 months, decreasing to 69.2% at 19 months and 53.1% at 24 months. Although the median follow-up duration was 12 months, a subset of patients had longer follow-up allowing Kaplan–Meier estimates up to 24 months.

## Discussion

In this real-world cohort of patients undergoing cryoballoon ablation for atrial fibrillation, we identified acute rhythm restoration strategy and simple echocardiographic parameters as clinically relevant factors associated with long-term AF recurrence. The principal findings of the study are threefold: first, AF recurrence occurred in approximately one-quarter of patients during follow-up; second, larger left atrial diameter and longer AF duration were strongly associated with recurrence; and third, successful restoration of sinus rhythm following standard direct current cardioversion was associated with a lower recurrence rate, suggesting that acute procedural rhythm response may reflect underlying atrial substrate rather than independently determining long-term outcomes.

Left atrial remodeling is a well-established substrate for AF persistence and recurrence [13]. Consistent with prior studies, patients with AF recurrence in our cohort exhibited significantly larger left atrial diameters [14, 15]. While left atrial diameter demonstrated only a borderline independent association with recurrence in multivariable analysis, its moderate discriminative performance in ROC analysis underscores its continued clinical relevance. Importantly, left atrial diameter represents a readily available and easily interpretable parameter, in contrast to more complex volumetric or strain-based indices that may not be routinely assessed in everyday practice [16, 17]. Left atrial size has been consistently identified as an independent preprocedural predictor of atrial fibrillation recurrence after catheter ablation, with a linear relationship reported between increasing left atrial anteroposterior diameter and the risk of post-ablation recurrence [18]. In our cohort, LAD showed a borderline association (*p* = 0.063) and moderate discrimination (AUC 0.692), indicating pragmatic but limited risk stratification value rather than a strong standalone predictor. In addition, higher body mass index was associated with AF recurrence in univariable comparisons, which is consistent with previous studies linking obesity to atrial remodeling and arrhythmia persistence. However, although BMI differed between groups in unadjusted comparisons, it was not independently associated with recurrence in logistic regression analysis, suggesting potential confounding by other clinical variables. A key and relatively underexplored finding of the present study is the association between acute rhythm restoration success and long-term outcomes. Successful conversion to sinus rhythm following standard DCCV was associated with a significantly lower recurrence rate. However, the univariable association of DCCV success with recurrence likely reflects underlying substrate severity and collinearity with AF type and AF duration rather than a causal effect, as this association was not maintained after multivariable adjustment and Cox time-to-event analysis. This observation suggests that acute rhythm response may act as a procedural surrogate marker reflecting both atrial substrate reversibility and immediate electrophysiological responsiveness to ablation [19]. Failure to maintain sinus rhythm despite acute intraprocedural cardioversion may reflect a more advanced atrial substrate, which could predispose patients to recurrent arrhythmia despite technically adequate pulmonary vein isolation [20].

The lack of independent association between amiodarone use and AF recurrence after adjustment likely reflects confounding by indication, as antiarrhythmic therapy is more frequently prescribed in patients with more advanced disease or higher perceived recurrence risk. Similarly, systolic pulmonary artery pressure did not emerge as an independent predictor in the multivariable model, suggesting that atrial structural and procedural factors may play a more dominant role in determining post-ablation rhythm outcomes in this population.

Our findings have several potential clinical implications. First, incorporation of simple echocardiographic measures and acute rhythm restoration outcomes into post-procedural assessment may facilitate early risk stratification. Second, patients with larger left atrial size or unsuccessful acute rhythm restoration may benefit from closer rhythm monitoring, earlier consideration of repeat intervention, or intensified adjunctive therapy. Rather than proposing a complex predictive score, this study emphasizes readily accessible markers that can be integrated into routine electrophysiology workflows without additional cost or specialized imaging.

Several limitations should be acknowledged. This was a single-center retrospective study conducted at a high-volume tertiary referral center with experienced operators, which may limit the generalizability of the findings, particularly to lower-volume centers. Left atrial diameter was used as a surrogate for atrial remodeling, whereas volumetric or functional assessments were not routinely available. AF type was not retained in the multivariable model due to quasi-complete separation, precluding assessment of its independent contribution after adjustment. Additionally, rhythm monitoring during follow-up was based on standard clinical practice and may have underestimated asymptomatic recurrences.

In addition, prolonged rhythm monitoring was not systematically performed, and therefore asymptomatic recurrences may have been underdetected. Furthermore, the complete-case Cox analysis excluded approximately 42% of patients due to missing covariates, which may have introduced selection bias.

Despite these limitations, the present study reflects real-world clinical practice with a substantial proportion of patients with persistent atrial fibrillation and provides clinically applicable insights into procedural and anatomical factors associated with AF recurrence after cryoballoon ablation.

## Conclusion

In a real-world cohort undergoing cryoballoon ablation for atrial fibrillation, acute cardioversion success was associated with recurrence in univariable analyses but was not independently predictive after multivariable adjustment. Left atrial diameter demonstrated moderate but limited discriminative ability. These findings suggest that cardioversion success reflects the underlying atrial substrate rather than exerting a causal effect on long-term outcomes, and that simple echocardiographic parameters may provide pragmatic but modest support for post-ablation risk stratification.

## Data Availability

The datasets generated and/or analyzed during the current study are available from the corresponding author on reasonable request.
